# Methylation of microRNA-338-5p by EED promotes METTL3-mediated translation of oncogene CDCP1 in gastric cancer

**DOI:** 10.18632/aging.103822

**Published:** 2021-04-21

**Authors:** Fangbin Zhang, Yan Yan, Xinguang Cao, Jinping Zhang, Yingxia Li, Changqing Guo

**Affiliations:** 1 Department of Gastroenterology, The First Affiliated Hospital of Zhengzhou University, Zhengzhou 450052, P. R. China; 2 Department of Oncology, The First Affiliated Hospital of Zhengzhou University, Zhengzhou 450052, P. R. China

**Keywords:** EED, methylation, microRNA-338-5p, METTL3, CDCP1

## Abstract

Unmasking the complex regulatory pathways that mediate the malignant phenotypes of cancer cells can provide novel targets for therapies that could limit the recurrence and metastasis of gastric cancer (GC). Herein, we intended to clarify the role of embryonic ectoderm development protein (EED), microRNA-228-5p (miR-338-5p), methyltransferase like 3 (METTL3) and CUB domain containing protein 1 (CDCP1) in GC. Differentially expressed miRNAs and their target genes were extracted by *in silico* analysis. The studies revealed high expression of EED in GC tissues and cell lines and it high expression in GC patients was shown to be associated with poor prognosis. The chromatin immunoprecipitation assay identified that EED methylated miR-338-5p to inhibit its expression. EED knockdown could restrain the proliferative and invasive abilities of GC cells by inducing miR-338-5p. Furthermore, miR-338-5p targeted m6A methylase METTL3, while METTL3 amplified the translation of CDCP1 *via* m6A activity which led to accelerated proliferation and invasion of GC cells. Moreover, *in vivo* experiments validated that EED promoted the progression of GC through mediating the miR-338-5p/METTL3/CDCP1 axis. Collectively, EED downregulated miR-338-5p through histone methylation, which in turn impaired miR-338-5p-dependent METTL3 inhibition and enhanced CDCP1 translation, therefore contributing to the development of GC.

## INTRODUCTION

Gastric cancer (GC) has been recognized as the second leading cancer-related death cause across the globe [1]. Notably, tumor biology and lymphatic spread of GC are much better understood, and treatments by surgical and palliative chemotherapeutic strategies have become more standardized [2, 3]. However, the treatment outcomes of GC are becoming unfavorable primarily due to the increased occurrence of metastatic events at advanced stages [4]. Recent studies have strongly supported the hypothesis that biomarker-specific treatment options have encouraging response rates and increased survival rates compared to traditional chemotherapy [5]. A better understanding of the signaling that influences the metastatic phenotypes can provide the information necessary to develop medicines to control and prevent advanced stages of the disease. Of note, dysregulation of the microRNA (miRNA) expression and its downstream effects on messenger RNA (mRNA) expression has been highlighted to possibly be implicated in GC progression [6]. Hence, identification of pathogenic mechanisms involving miRNA/mRNA in GC progression may contribute to developing more alternative therapeutic targets for this malignancy.

The tumor suppressive role of miR-338-5p has been well documented by previous studies. For example, a low expression of miR-338-5p has been noted in colorectal carcinoma and its ectopic expression enhanced sensitivity of cancer cells to oxaliplatin [7]. Another study on glioblastomas have established the tumor-inhibiting properties of miR-338-5p by proving the restricted proliferative and invasive activities of glioblastoma cells [8]. Furthermore, DNA methylation of miRNAs in GC has been extensively highlighted in recent research which offer invaluable insight on the functional relevance of epigenetic mediation of miRNAs leading to GC pathogenesis [9].

Of interest, the murine polycomb group protein embryonic ectoderm development (EED) is responsible for global histone H3 lysine-27 trimethylation (H3K27me3) [10]. Enhancer of zeste homolog 2 (EZH2) and EED have been determined as the two key catalytic subunits of polycomb repressive complex 2 which is involved in the epigenetic regulation associated with the progression of GC [11]. The tumor suppressive effects be realized through selective repression of H3K27me3 by concentration-dependently blocking the EZH2/EED axis and diminishing EZH2 levels [12]. A prior study tried to discover an EED inhibitor with anti-cancer efficacy, and suggested that specific and direct inhibition of EED can result in tumor repression [13]. More importantly, increased EZH2 expression have been suggested to play a part in epigenetic silencing of miR-338-5p [14]. In the current study, it has been established that methyltransferase like 3 (METTL3) is the target gene of miR-338-5p. METTL3 is a critical factor associated with the large N6-adenosine-methyltransferase complex which in mammalian is required for N6-methyladenosine (m6A) modification among a wide array of miRNAs [15]. Additionally, m6A methylase METTL3 was found to accelerate translation of oncogenes in lung cancer and consequently enhancing the growth, survival, and invasion of cancer cells [16]. Moreover, METTL3 has been highlighted to augment the m6A modification in 3’-untranslated region (3'-UTR) of CUB domain containing protein 1 (CDCP1) mRNA, which is positively correlated with the malignancy status of bladder cancer [17]. This study aimed to outline new mechanisms by which EED-mediated miR-338-5p/METTL3/CDCP1 axis exert oncogenic effects in GC initiation and development.

## RESULTS

### Elevated expression of EED and its association with poor prognosis in GC

**Figure 1:**
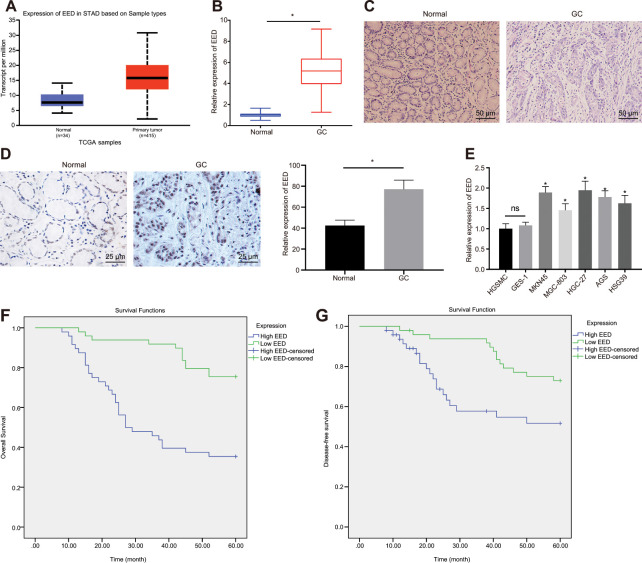
**EED is highly expressed in GC tissues and cells.** (**A**) Absolute EED expression in GC obtained by UALCAN database (http://ualcan.path.uab.edu/index.html). The blue box on the left represents the expression of normal samples, and the red box on the right represents the expression of GC samples. (**B**) RT-qPCR determination of EED expression in GC tissues and adjacent normal tissues (n = 97). (**C**) Representative images of GC tissues and adjacent normal tissues by HE staining. (**D**) Immunohistochemistry to assess the EED expression in GC tissues and adjacent normal tissues. (**E**) RT-qPCR to examine EED expression in GC cell lines and normal gastric cell lines, with β-actin as internal control. (**F**, **G**) Kaplan-Meier method with log-rank test to assess overall survival (**F**) and disease-free survival (**G**) of patients with relatively higher or lower EED expression (n = 97). Measurement data are expressed as mean ± standard deviation. * *p* < 0.05 compared with normal gastric cell line. GC tissues were compared with adjacent normal tissues by paired *t* test, n = 97. Data comparison among multiple groups was performed using one-way ANOVA with Tukey's post hoc test. Kaplan-Meier method was carried out to investigate the relationship between high and low expression of EDD in GC tissues and overall survival and disease-free survival (log-rank test). Cell experiments were repeated 3 times independently.

The EED expression in GC was analyzed in UALCAN database (http://ualcan.path.uab.edu/index.html). The UALCAN analysis revealed a high expression of EED in GC (Figure 1A). Reverse transcription-quantitative polymerase chain reaction (RT-qPCR) further confirmed elevated EED expression in GC tissue in comparison to adjacent normal tissue (Figure 1B). The hematoxylin-eosin (HE) staining (Figure 1C) and immunohistochemical analysis (Figure 1D) validated elevated EED expression in GC tissue localized in the nucleus. Subsequently, the expression of EED in human gastric smooth muscle cells (HGSMCs) was not found to be different from that in GES-1 cells, however it was lower than that in GC cell lines (MKN45, MGC-803, HGC-27, AGS and HSG39) (Figure 1E). Furthermore, both the disease-free survival and overall survival of patients with high expression of EED were shortened (Figure 1F, 1G). This suggests that elevated expression of EED in GC is related to poor prognosis.

### EED downregulates the expression of miR-338-5p

**Figure 2:**
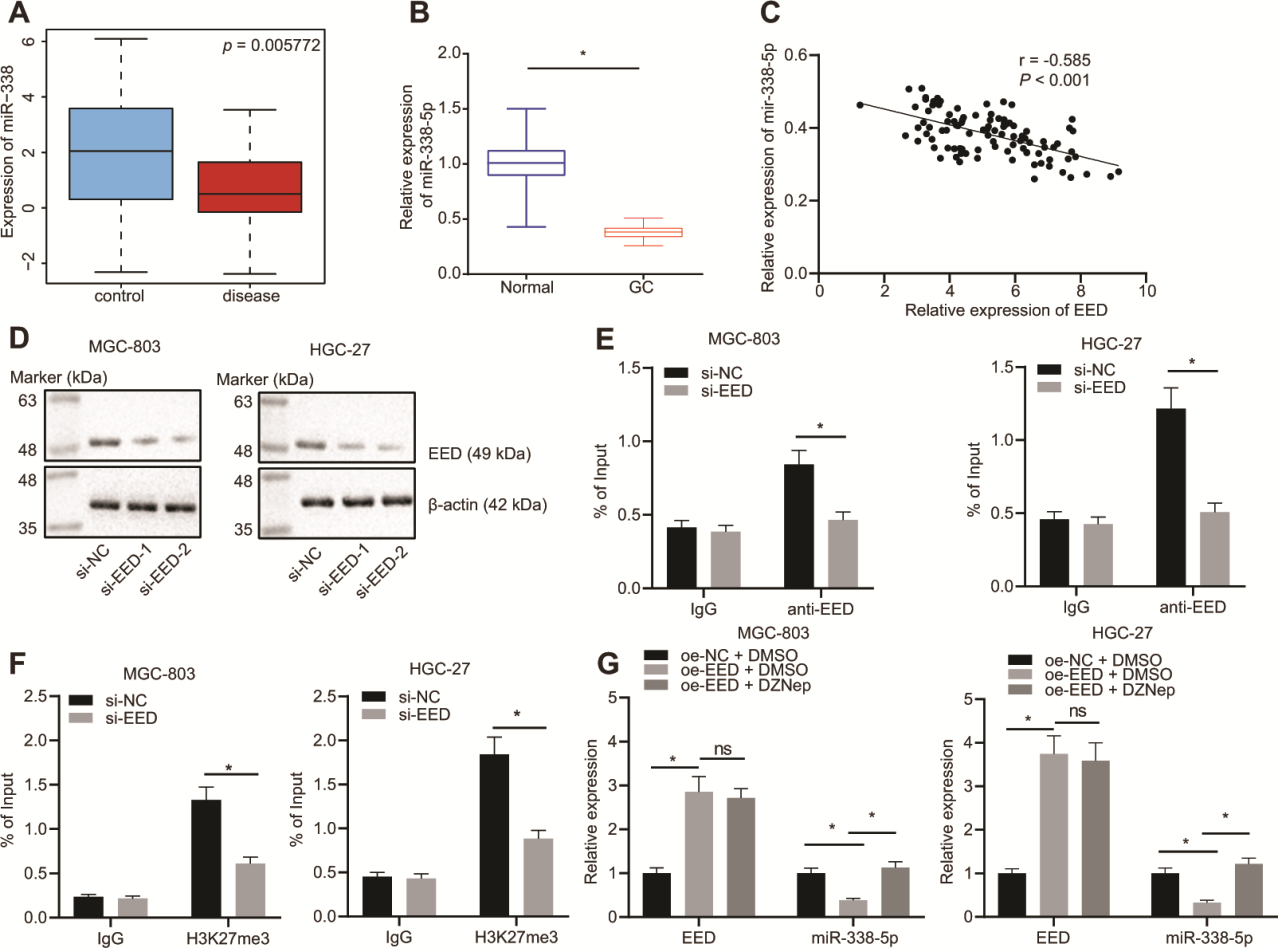
**EED inhibits miR-338-5p expression by promoting methylation.** (**A**) Absolute miR-338-5p expression in profile GSE23739 obtained from GEO database (https://www.ncbi.nlm.nih.gov/gds) analyzed by R language. The blue box on the left represents the expression of normal samples, and the red box on the right represents the expression of GC samples (*p* = 5.772E-03). (**B**) RT-qPCR determination of miR-338-5p expression in GC tissues and adjacent normal tissues (n = 97). (**C**) miR-338-5p expression in GC tissues was negatively correlated with EED expression in GC tissues (n = 97). (**D**) Evaluation of EED knockdown efficiency in MGC-803, and HGC-27 cells. (**E**) ChIP assay to assess enrichment of EED in the miR-338-5p promoter. (**F**) Enrichment of H3K27me3 in the miR-338-5p promoter. (**G**) RT-qPCR to examine EED and miR-338-5p expression after overexpressing EED and DZNep treatment, with β-actin and U6 as internal control, respectively. Measurement data are expressed as mean ± standard deviation. * *p* < 0.05. GC tissues were compared with adjacent normal tissues by paired *t* test, and other comparison between two groups was analyzed using an unpaired *t* test. Data comparison among multiple groups was performed using one-way ANOVA with Tukey's post hoc test. Pearson’s correlation analysis was carried out for the correlation between miR-338-5p and EED expression in GC tissues. Cell experiments were repeated 3 times independently.

The Gene Expression Omnibus (GEO) database profile GSE23739 (Figure 2A) identified decreased expression of miR-338-5p in GC. We also confirmed that miR-338-5p was poorly expressed in GC tissues (Figure 2B) and was negatively correlated to EED expression (Figure 2C). A small interfering RNA (siRNA) sequence was designed specifically for EED (Figure 2D). This siRNA targeting EED (si-EED) showed high knockdown efficiency in MGC-803 and HGC-27 cells, which was also employed in subsequent experiments. The chromatin immunoprecipitation (ChIP) assay revealed that EED was enriched in the miR-338-5p promoter region (Figure 2E). Additionally, the H3K27me3 marker was also enriched in the miR-338-5p promoter region, and the enrichment decreased after knocking down EED (Figure 2F), which indicated that EED can bind to the miR-338 promoter region and augment its methylation. In response to overexpression of EED in MGC-803 and HGC-27 cells, we observed that EED expression was increased while miR-338-5p expression was reduced. Protein methyltransferase inhibitor 3-Deazaneplanocin A (DZNep) is an inhibitor of histone methylation. EED expression did not differ after histone methylation was suppressed by DZNep treatment; however, miR-338-5p expression was elevated (Figure 2G), which suggested that EED inhibits miR-338-5p expression by promoting histone methylation.

### EED knockdown impedes the proliferation and invasion of GC cells by inducing miR-338-5p

**Figure 3:**
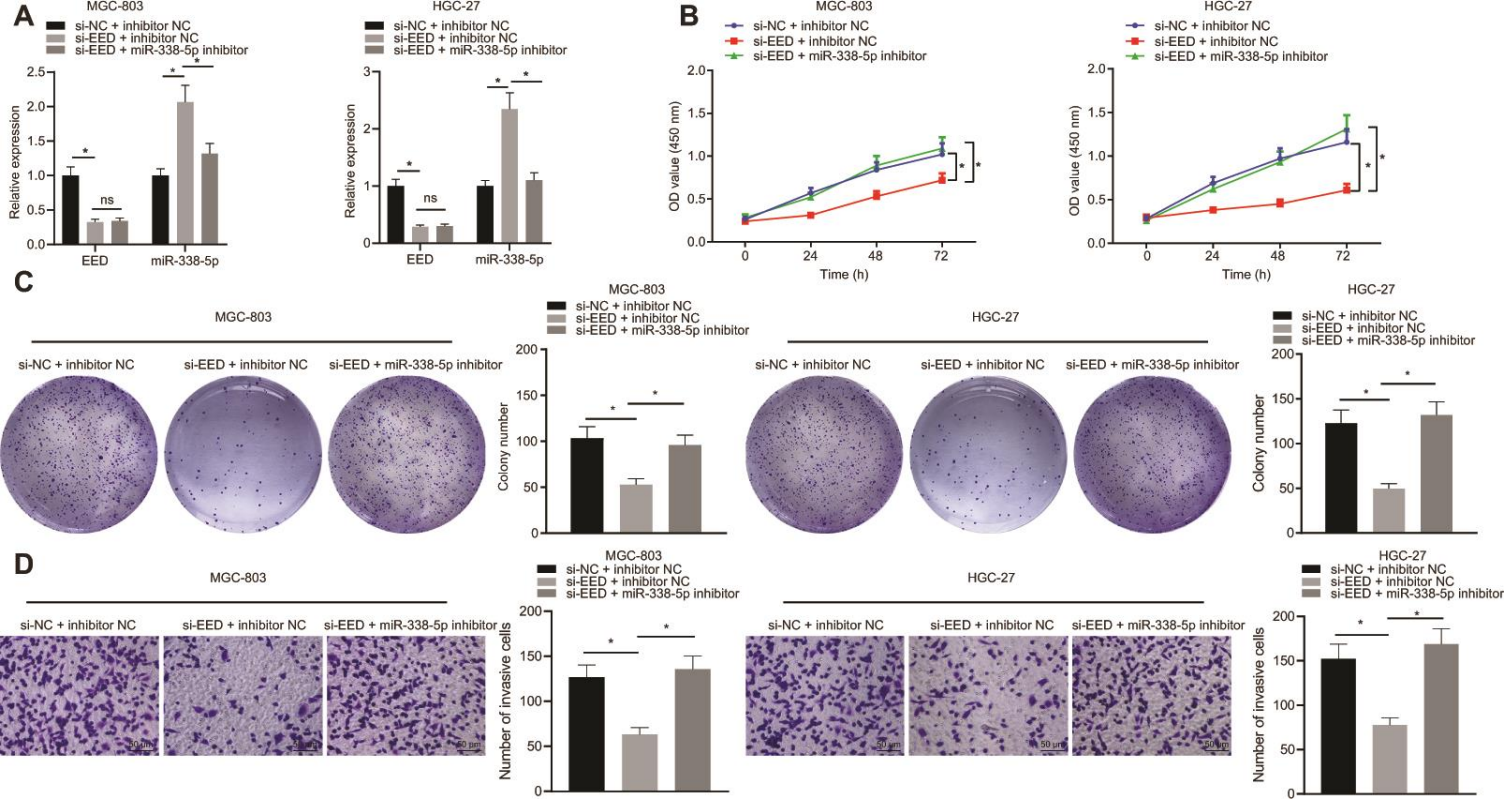
**EED silencing upregulates miR-338-5p to restrain the proliferation and invasion of GC cells.** (**A**) RT-qPCR to examine EED, miR-338-5p expression in response to si-EED and miR-338-5p inhibitor in MGC-803 and HGC-27 cells, with β-actin and U6 as internal control, respectively. (**B**) CCK-8 assay to assess cell viability in response to si-EED and miR-338-5p inhibitor. (**C**) Colony formation assay to assess the cell colony formation in response to si-EED and miR-338-5p inhibitor. (**D**) Transwell assay to assess the number of invaded cells in response to si-EED and miR-338-5p inhibitor. Measurement data are expressed as mean ± standard deviation. * *p* < 0.05. Data comparison among multiple groups was performed using one-way ANOVA with Tukey's post hoc test. Data comparison between groups at different time points was performed using two-way ANOVA or repeated-measures ANOVA with Bonferroni post hoc test. Cell experiments were repeated 3 times independently.

In MGC-803 and HGC-27 cells, knockdown of EED effectively augmented miR-338-5p expression. Additionally, miR-338-5p expression effectively inhibited after transfection with miR-338-5p inhibitor, which did not affect EED expression (Figure 3A). Experiments performed using cell counting kit-8s (CCK-8) and colony formation assays determined that GC cell proliferation was reduced after EED knockdown. Furthermore, Transwell assay further revealed that cell invasion was also suppressed following EED knockdown. However, after miR-338-5p inhibitor restricted miR-338 expression, cell proliferation and invasive ability were restored in the presence of EED knockdown (Figure 3B–3D), which indicated that downregulated EED enhanced miR-338-5p expression which consequently suppressed the proliferation and invasion of GC cells.

### miR-338-5p targets m6A methylase METTL3

**Figure 4:**
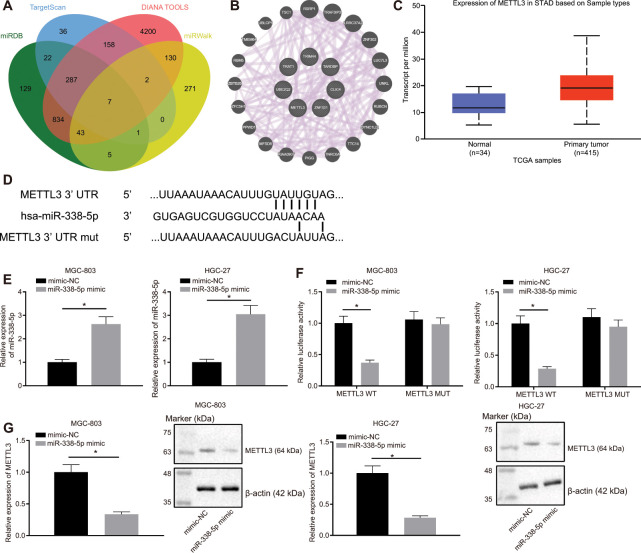
**miR-338-5p targets the m6A methylase METTL3.** (**A**) Venn map of the downstream genes of miR-338-5p predicted by the websites miRDB (http://www.mirdb.org/, Target Score ≥ 50), TargetScan (http://www.targetscan.org/vert_71/, cumulative weighted context ++ score < -0.15), DIANA TOOLS (http://diana.imis.athena-innovation.gr/DianaTools, miTG score > 0.45) and miRWalk (http://mirwalk.umm.uni-heidelberg.de/; energy < -23, accessibility < 0.01, au > 0.45). (**B**) The PPI network of seven miR-338 downstream genes and their related genes constructed by GeneMANIA (http://genemania.org/). Larger circle of the related genes in the figure reflects higher core level, otherwise reflects lower core level. (**C**) Absolute METTL3 expression in GC obtained by UALCAN analysis. The blue box on the left represents the expression of normal samples, and the red box on the right represents the expression of GC samples. (**D**) TargetScan to predict the binding sites of miR-338-5p to METTL3. (**E**) RT-qPCR determination of miR-338-5p expression in MGC-803 and HGC-27 cells in response to miR-338-5p mimic, with U6 as internal control. (**F**) Dual-luciferase reporter gene assay to detect luciferase activity in the presence of miR-338-5p mimic. (**G**) RT-qPCR and Western blot assay to examine METTL3 expression after transfection with miR-338-5p mimic, with β-actin as internal control. Measurement data are expressed as mean ± standard deviation. * *p* < 0.05. Unpaired *t* test was employed for data comparison between two groups, and cell experiments were repeated 3 times independently.

Through the websites miRDB, TargetScan, DIANA TOOLS, and miRWalk, we predicted downstream genes of miR-338-5p and obtained 1328, 513, 5661, and 459 downstream genes, respectively. In the intersection of the results from the four databases, we identified seven important downstream genes (Figure 4A). Subsequently, GeneMANIA was employed to construct a protein-protein interaction (PPI) network of these seven genes, which determined METTL3 was the second core gene (Figure 4B and Table 1). An existing literature has revealed that METTL3 is a m6A methylase [18], which regulates the downstream pathway *via* m6A methylase. UALCAN analysis confirmed that METTL3 was highly expressed in GC (Figure 4C). Furthermore, TargetScan predicted the binding sites of human and mouse miR-338-5p to METTL3 (Figure 4D). Moreover, MGC-803 and HGC-27 cells were transfected with the luciferase reporter plasmid METTL3-3'-UTR wild type (WT) or METTL3-3'-UTR mutant (MUT), and miR-338 mimic. This experiment showed that the miR-338 mimic can effectively elevate the miR-338 expression (Figure 4E). Additionally, we also found that miR-338 mimic effectively inhibited the luciferase activity of METTL3-3'-UTR WT, whereas had no obvious effect on METTL3-3'-UTR MUT (Figure 4F). It was suggested that miR-338 could bind to the METTL3-3'-UTR and suppress METTL3 expression. When miR-338 mimic was transfected into MGC-803 and HGC-27 cells, the mRNA and protein expression levels of METTL3 were also reduced (Figure 4G), confirming that miR-338 bound to METTL3 and restricted its expression.

**Table 1:** Core degrees of genes in protein-protein interaction network.

**Rank**	**Gene**	**Degree**
1	TARDBP	19
2	METTL3	17
3	UBE2Q2	4
4	TRAT1	1
5	CLIC4	1
6	TRIM44	1
7	ZNF331	0

Note: Degree indicates the number of the interaction between the gene and other genes, also referring to core degree.

### METTL3 elevates CDCP1 expression through m6A to accelerate GC cell proliferation and invasion

**Figure 5:**
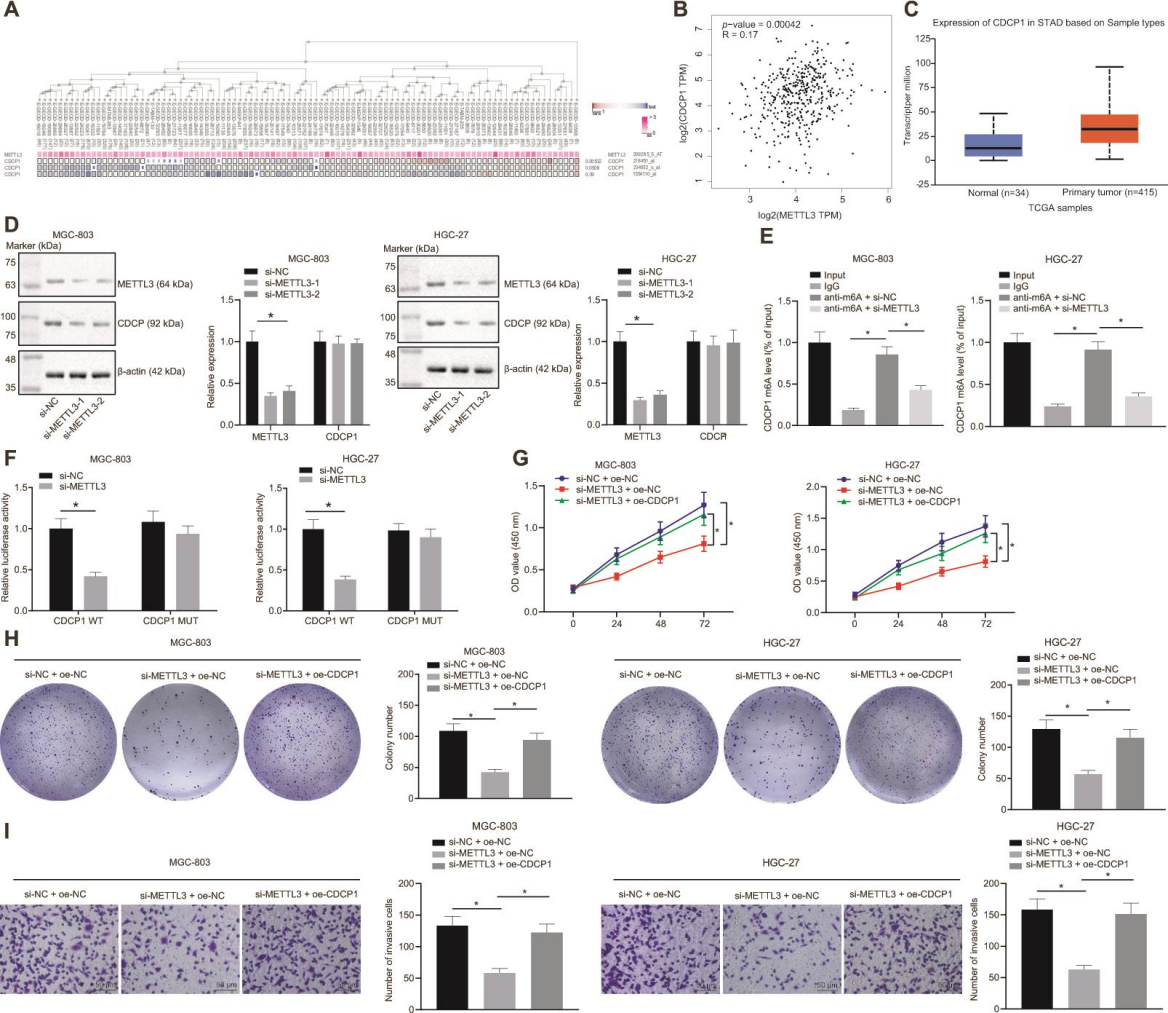
**METTL3 modifies CDCP1 through m6A to elevate the CDCP1 expression, driving the proliferation and invasion of GC cells.** (**A**) The co-expression map of METTL3 and CDCP1 obtained by MEM analysis (*p* = 5.32E-03; https://biit.cs.ut.ee/mem/index.cgi). (**B**) Correlation between METTL3 and CDCP1 expression obtained by GEPIA analysis (http://gepia2.cancer-pku.cn) of GC data (R = 0.17, *p* = 4.2E-04), absolute expression of mRNAs from GEPIA is presented. (**C**) Absolute CDCP1 expression in GC obtained by UALCAN. The blue box on the left represents the expression of normal samples, and the red box on the right represents the expression of GC samples. (**D**) Western blot assay and RT-qPCR to measure METTL3 and CDCP1 expression in MGC-803 and HGC-27 cells, with β-actin as internal control. (**E**) Me-RIP assay to examine m6A level of CDCP1 mRNA and enrichment after METTL3 knockdown. (**F**) Quantitative analysis for luciferase activity after METTL3 knockdown. (**G**) CCK-8 assay for cell viability in response to METTL3 knockdown and CDCP1 overexpression. (**H**) Colony formation assay to assess the number of colonies in response to METTL3 knockdown and CDCP1 overexpression. (**I**) Transwell assay to assess the number of invaded cells in response to METTL3 knockdown and CDCP1 overexpression. Measurement data are expressed as mean ± standard deviation. * *p* < 0.05. Unpaired *t* test was employed for data comparison between two groups. Data comparison among multiple groups was performed using one-way ANOVA with Tukey's post hoc test. Data comparison between groups at different time points was performed using two-way ANOVA or repeated-measures ANOVA with Bonferroni post hoc test. Cell experiments were repeated 3 times independently.

By employing the MEM analysis, we established a significant co-expression relationship between METTL3 and CDCP1 (Figure 5A). The Gene Expression Profiling Interactive Analysis (GEPIA) further suggested that METTL3 was positively correlated with CDCP1 expression (Figure 5B), and the UALCAN analysis found that CDCP1 was highly expressed in GC (Figure 5C). Subsequently, we knocked METTL3 down in MGC-803 and HGC-27 cells, and the test showed no significant change in the mRNA expression of CDCP1 but its protein expression was reduced (Figure 5D). The Me-RNA binding protein immunoprecipitation (Me-RIP) assay revealed that the m6A level of CDCP1 mRNA was increased, which was consequently diminished following the knockdown of METTL3 (Figure 5E). The dual luciferase reporter plasmid CDCP1-3'-UTR WT and CDCP1-3'-UTR-mut (m6A motif) was constructed. It was also demonstrated that the luciferase activity of CDCP1-3'-UTR WT was reduced after knocking down METTL3, whereas no significant change was witnessed after the mutation of the m6A motif (Figure 5F). These results indicated that METTL3 enhanced the m6A modification of CDCP1 mRNA and augmented its transcription expression in MGC-803 and HGC-27 cells. Besides, we found that compensating the expression of CDCP1 after the knockdown of METTL3 in MGC-803 and HGC-27 cells can effectively boost the proliferation and invasion of GC cells (Figure 5G–5I). This confirmed that METTL3 could accelerate GC cell proliferation and invasion by elevating CDCP1 expression.

### EED accelerated GC progression *in vivo* through the miR-338-5p/METTL3/CDCP1 axes

**Figure 6:**
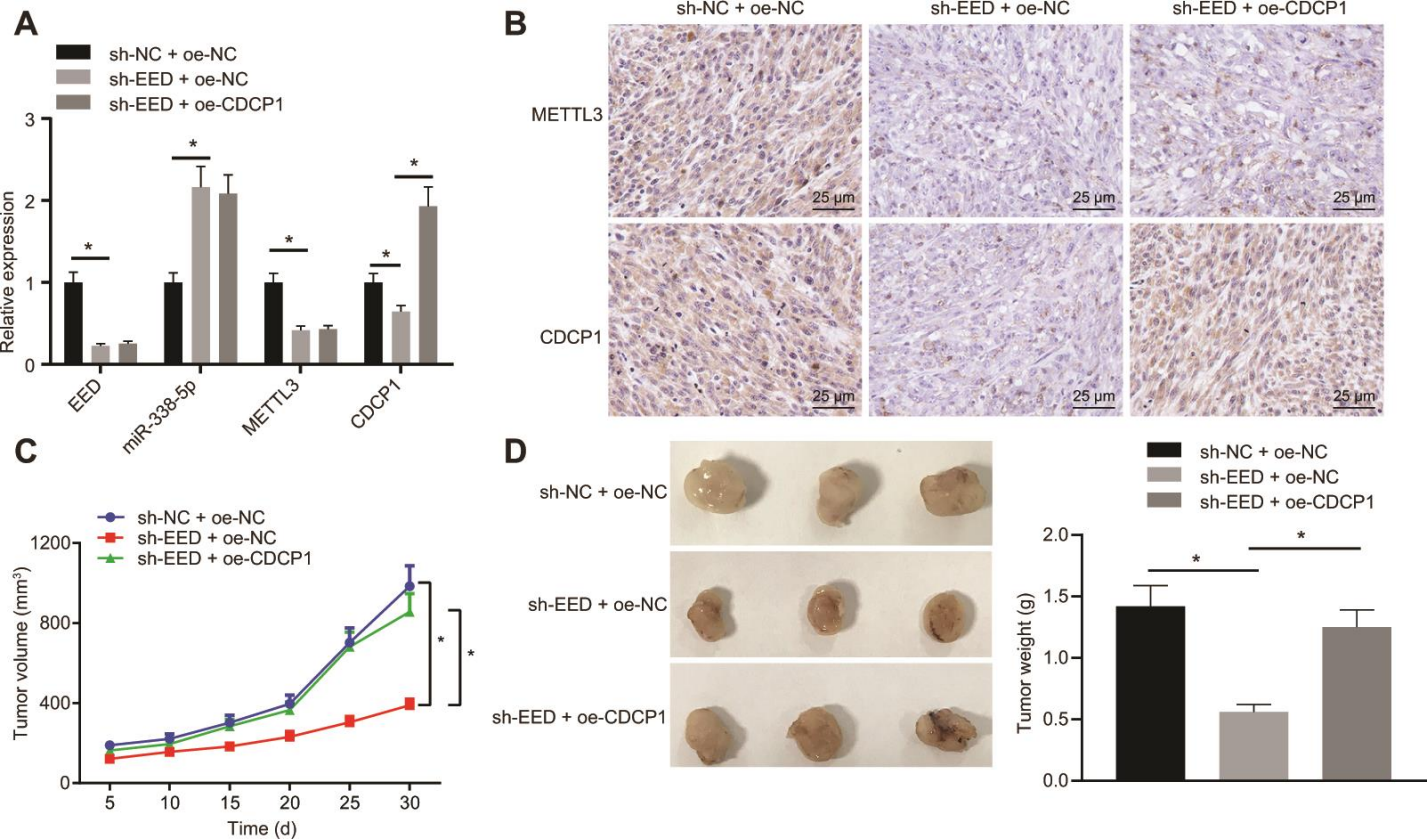
**EED accelerates the progression of GC *in vivo* through the miR-338-5p/METTL3/CDCP1 axis.** (**A**) RT-qPCR to examine the expression of EED/miR-338-5p/METTL3/CDCP1 in tumors of nude mice, with β-actin and U6 as internal control, respectively. (**B**) Immunohistochemistry to detect METTL3 and CDCP1 expression in mouse tumors. (**C**) Tumor volume and tumor photos in response to EED knockdown and CDCP1 overexpression. (**D**) Quantitative analysis for tumor weight in response to EED knockdown and CDCP1 overexpression. Measurement data are expressed as mean ± standard deviation. * *p* < 0.05. Data comparison among multiple groups was performed using one-way ANOVA with Tukey's post hoc test. Data comparison between groups at different time points was performed using two-way ANOVA or repeated-measures ANOVA with Bonferroni post hoc test. n = 6.

On the basis of the results of the bioinformatic investigations, we established that EED mediated m6A methylase METTL3 and CDCP1 through miR-338-5p, thereby affecting the progression of GC. The nude mice were subcutaneously injected with MGC-803 cells which were stably transduced with lentivirus-packaged short hairpin RNA (sh)-negative control (sh-NC) + overexpression (oe)-NC, sh-EED + oe-NC, or sh-EED + oe-CDCP1. The RT-qPCR analysis revealed that the expression of METTL3 and CDCP1 was reduced, whereas the expression of miR-338-5p was elevated after knocking down EED (Figure 6A). Meanwhile, CDCP1 overexpression did not show any significant effect on the expression of EED, METTL3 or miR-338-5p. The immunohistochemistry assay revealed that METTL3 and CDCP1 expression was diminished after the knockdown of EED while overexpression of CDCP1 did not affecting the expression of METTL3 (Figure 6B). The cumulative data indicated that EED inhibited miR-338-5p expression *in vivo*, thereby promoting both the expression of m6A methylase METTL3 and the m6A modification of CDCP1. Measurement of tumor volume in mice from the 5^th^ day showed slowed tumor growth (Figure 6C) and tumor weighting after euthanasia on the 30^th^ day of inoculation revealed significant reduced tumor weight after EED knockdown (Figure 6D). However, both tumor volume and weight were restored after overexpression of CDCP1. It shows that EED accelerates GC occurrence through miR-338-5p/METTL3/CDCP1 axis.

## DISCUSSION

**Figure 7:**
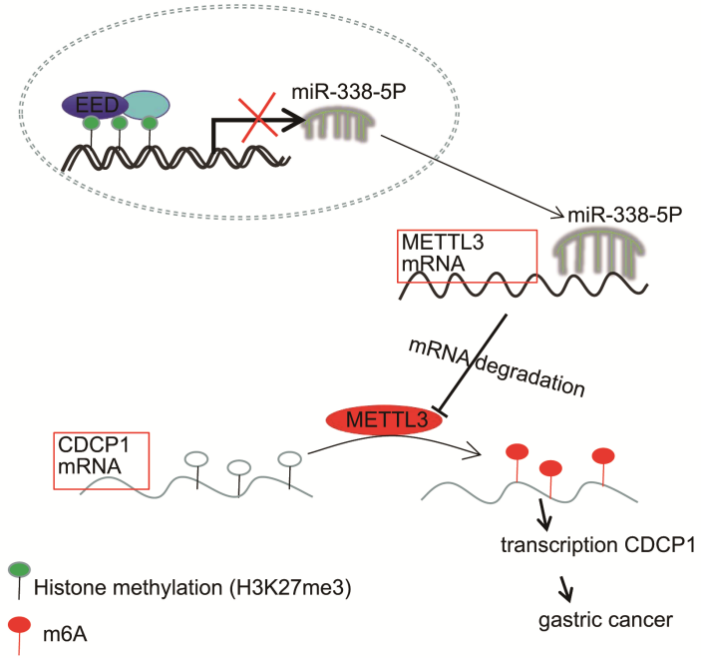
**The mechanism graph of the regulatory network and function of EED-mediated miR-338-5p methylation.** EED methylates miR-338-5p to repress its expression, and upregulates expression of miR-338-5p’s target METTL3. METTL3 modifies CDCP1 mRNA through m6A to promote CDCP1 translation, leading to GC progression.

Accumulating evidence has demonstrated the molecular mechanisms of tumorigenesis in the stomach, through investigation and evaluation of which, the identification of novel targets may offer a promising perspective for the treatment of GC which, at present, remains incurable [19]. Previous researchers have unmasked the critical histone methyltransferase activity of the EED-EZH2 complex and related the function of this complex to cancer metastasis [20]. More interestingly, the proliferative and invasive potential of tumor cells can be restricted through disrupting the aforementioned EZH2-EED complex [12]. Other studies have addressed that the positive correlations between EZH2 and miRNAs, for example miR-181b in prostate cancer and miR-101-3p in GC, have been indicated to have tumor suppressive properties at molecular levels [21, 22]. The current study, aimed to illuminate the molecular mechanisms through which the EED-mediated miR-338-5p/METTL3/CDCP1 axis fine-tune the malignant phenotypes of GC cells (Figure 7).

The experimental data unveiled that EED was frequently overexpressed in GC and its increased expression was often associated with poor prognosis. Interestingly, existing evidence also revealed that the dysfunction of the PRC2 complex comprising of subunits such as EZH2, SUZ12, and EED could expedite abnormal hypermethylation of H3K27 which induces carcinogenesis in a wide array of cancers. Furthermore, the ubiquitin-mediated protein degradation of EED was shown to restrict proliferative potential of cancer cells [23]. Liu et al*.* also identified higher expression of EED in cancerous tissues, relative to non-cancerous tissue, in colorectal cancer. Moreover, they observed the positive correlation between EED overexpression and the aggressiveness of the clinicopathological features of patients with colorectal cancer, such as facilitated lymph node metastasis, distal metastasis and lower disease-free survival [24]. Therefore, we strongly supported the hypothesis that EED may be involved in the pathogenesis and progression of GC.

We then proceeded to explain the underlying mechanisms and found that EED methylated miR-338-5p and inhibited miR-338-5p expression, and enhanced the proliferative and invasive ability of GC cells. Prior evidence has indicated that miR-338-5p was proposed to be a biomarker for the diagnosis of hepatocellular carcinoma owing to its negative correlation with the level of AFP [25]. In another study on glioblastoma multiforme, the overexpression of miR-338-5p was found to diminish the cell proliferative activities and to facilitate cell cycle arrest and apoptosis [26]. Additionally, ectopic expression of miR-338 has been shown to restrict the proliferative activities and epithelial-mesenchymal transition of lung cancers, which was realized through downregulating its target NFATc1 [27]. Hence, we speculated that the tumor suppressive function of miR-338-5p may also be achieved by mediating target genes.

Computer-based miRNA target detection programs were employed to predict the potential target genes of miR-338-5p, which revealed that the m6A methylase METTL3 was a core candidate target of miR-338-5p. This was further confirmed by using the dual-luciferase reporter gene assay. METTL3 is an RNA methyltransferase capable of orchestrating m6A modification, which has an important role to play in mRNA biogenesis and translation [28]. In a previous study of brain metastasis of lung cancer, METTL3 was found to intensify the splicing of miR-143-3p therefore elevating its biogenesis in raising the cell motility [29]. A higher level of m6A mRNA methylation initiated by METTL3 facilitates YAP mRNA translation leading to an acceleration of the invasive and metastatic activities of non-small cell lung cancer cells [30]. The mechanistic investigation of this study further clarified that METTL3 modified CDCP1 through m6A and elevated its expression, whereby driving the malignant phenotypes of GC cells. Another previous study corroborated our findings, which revealed that both METTL3 and CDCP1 were robustly expressed in bladder cancer, and also that the suppression of the METTL3-m6A-CDCP1 axis could restrict the bladder cancer growth and tumorigenesis both *in vitro* and *in vivo* [17]. Moreover, the tumor initiating role of CDCP1 has been identified in GC as evidenced by its function in accelerating migratory and invasive potential of cancer cells [31]. Our *in vivo* findings validated the *in vitro* results and revealed that EED enhanced GC progression *in vivo* through the miR-338-5p/METTL3/CDCP1 axis.

In our current work, we validated the oncogenic function of EED in reinforcing the proliferative, and invasive capacities of GC cells through a series of functional experiments conducted both *in vitro* and *in vivo*. Mechanistically, EED methylates miR-338-5p to repress its expression, and upregulates expression of miR-338-5p’s target METTL3. Further METTL3 modifies CDCP1 mRNA through m6A to promote the translation of CDCP1. As we further elucidate the epigenetic regulatory mechanisms underlying GC, further experiments and clinical trials are necessary for translational applications for patients with GC.

## MATERIALS AND METHODS

### Ethics statement

The study was approved by the Ethics Committee of the First Affiliated Hospital of Zhengzhou University. Signed informed consents were obtained from all participating patients. All animal experiments were conducted following the protocol approved by the Laboratory Animal Care and Use Committee of the First Affiliated Hospital of Zhengzhou University. And great efforts were made to minimize the suffering of the included animals.

### Patient enrollment

From January 2011 to January 2014, 97 patients (61 males and 36 females) with GC were randomly selected in the First Affiliated Hospital of Zhengzhou University. The patients aged 49 - 72 years, with an calculated average age of 60.38 ± 5.22 years. None of the patients had received radiotherapy or chemotherapy before experimentation. All tissue samples were immediately placed in liquid nitrogen after extraction and then stored at -80° C. In addition, patients were followed up until January 2019 and regularly monitored for a follow-up period of 60 months.

### Cell culture

HGSMCs and human GC cells (MKN45 and MGC-803) were cultured in 90% Dulbecco's Modified Eagle's Medium-High Glucose (DMEM-H) which contained 10% fetal bovine serum (FBS). Human gastric mucosal cells (GES-1) and human GC cells (HGC-27, AGS, and HSG39) were cultured in 90% Roswell Park Memorial Institute-1640 medium which also contained 10% FBS. Further a penicillin-streptomycin (1 : 1) solution was added, with 100 U/mL as the final concentration, and the cells were then cultured in an incubator at the temperature of 37° C along with 5% CO_2_. All cell lines were purchased from American Type Culture Collection (Manassas, VA, USA).

### RNA extraction and RT-qPCR

Total RNA was extracted using TRIzol following the manufacturer's protocol. For mRNA detection, RT was performed using a RT kit (RR047A, Takara, Japan) to obtain cDNA. For miRNA detection, the cDNA was obtained by RT using the Mir-XTM miRNA First Strand Synthesis Kit (Takara, Dalian, Liaoning, China). Quantification of miRNA level was checked by the Mir-XTM miRNA real time qPCR TB GreenTM Kit (Takara). Samples were loaded by employing the SYBR® Premix Ex TaqTM II (Perfect Real Time) kit (DRR081, Takara), and furthermore the samples were analyzed for mRNA expression using specific primers in the ABI 7300 qPCR system. The relative expression level of mRNA was normalized to endogenous glyceraldehyde-3-phosphate dehydrogenase (GAPDH) whereas the relative expression level of miRNA was normalized to U6 (Table 2). The relative expression of the product was calculated using the 2^-ΔΔCt^ method.

**Table 2:** Primer sequences for RT-qPCR.

**Gene**	**Primer sequence (5'-3')**
EED	F: 5'-GTGTGCGATGGTTAGGCG-3'
	R: 5'-GTCACATTAGATTCACTGGGTTT-3'
miR-338-5p	F: 5'-ATCCAGTGCGTGTCGTG -3'
	R: 5'-TGCTAACAATATCCTGGTG-3'
METTL3	F: 5'-CAA CAT ACC CGT ACT ACA GGA-3'
	R: 5'-TTC ATC TAC CCG TTC ATA CCC -3'
CDCP1	F: 5'- CTGAACTGCGGGGTCTCTATC-3'
	R: 5'-GTCCCCAGCTTTATGAGAACTG -3'
GAPDH	F: 5'-GCACCGTCAAGGCTGAGAAC-3'
	R: 5'-TGGTGAAGACGCCAGTGGA-3'
U6	F: 5'-GCTTCGGCAGCACATATACTAAAAT-3'
	R: 5'CGCTTCACGAATTTGCGTGTCAT-3'

Note: RT-qPCR, reverse transcription-quantitative polymerase chain reaction; F, forward; R, reverse; EED, embryonic ectoderm development protein; miR, microRNA; METTL3, methyltransferase like 3; COCP1, CUB domain containing protein 1; GAPDH, glyceraldehyde-3-phosphate dehydrogenase.

### Cell transfection and dual-luciferase assay

The plasmids si-EED, si-METTL3 were purchased from GenePharma (Shanghai, China). Lipofectamine^TM^ 2000 Transfection Reagent (11668019, Invitrogen, Carlsbad, CA, USA) was employed for transfection according to the instructions. RT-qPCR and Western blot assay were performed after 48 h. The METTL3 3'-UTR was artificially synthesized, and the complementary sequence mutation site fragment METTL3 3'-UTR-MUT of the seed sequence was designed on the wild type. Next the METTL3 3'-UTR, and m6A mutant sequences were constructed into a pMIR-reporter plasmid. The WT or MUT plasmids were co-transfected into cells with miR-183 mimic or mimic NC in accordance with the Lipofectamine™ 2000 instructions. Following the transfection, the cells were cultured for 24 h, and then the luciferase activity of each group was measured using the Promega's Dual-Luciferase Reporter Assay System (Promega, Madison, WI, USA).

### Immunoblotting assay

Total protein in tissues or cells was extracted with a Radio Immunoprecipitation Assay lysis buffer solution containing phenylmethyl sulfonylfluoride. Cells were then centrifuged at a temperature of 4° C and at the rate of 8000 ×g for the duration of 10 minutes to harvest the supernatant. The bicinchonininc acid kit (P0012, Beyotime Institute of Biotechnology, China) was employed to measure the total protein concentration. Then, 50 μg of protein was dissolved in 2 × sodium dodecyl sulfate (SDS) loading buffer. After boiling both at 100° C for 10 min, each sample was subjected to SDS-polyacrylamide gel electrophoresis. The protein was transferred to a polyvinylidene fluoride (PVDF) membrane using wet transfer. After blocking the PVDF membrane for 1 h, it was incubated at 4° C overnight with diluted primary antibodies: rabbit anti-EED (1 : 1000, PA5-34430, Invitrogen), rabbit anti-METTL3 (1 : 1000, ab195352, Abcam, Cambridge, UK), rabbit anti-CDCP1 (1 : 1000, ab1377, Abcam), and murine anti-β-Actin (1 : 5000, ab8227, Abcam). This was followed by incubation with secondary antibody to immunoglobulin G (IgG) (Abcam, ab205718, goat anti-rabbit, 1 : 20000; Abcam, ab205719, goat anti-mouse, 1 : 20000) at room temperature for 1 h. The blots were developed with the enhanced chemiluminescence substrate (WBKLS0100, Millipore, Billerica, MA, USA) and quantified using the Image J software.

### Immunohistochemistry

To begin, the tissue sections were dewaxed and hydrated. Then the antigens were retrieved in a citrate repair solution. The tissue sections were subjected to blocking in Tris buffered saline solution containing 10% normal serum and 1% bovine serum albumin for 2 h at room temperature. Each section was then treated with EDD antibody (1 : 100, PA5-34430, Invitrogen), CDCP1 (1 : 100, ab1377, Abcam), and METTL3 (1 : 500, ab195352, Abcam) at 4° C overnight. Normal serum was used as the NC instead of using a primary antibody. Then, 50 μL of 3% H_2_O_2_ was added to each section, which was followed by incubation at room temperature for 20 min with the purpose of eliminating endogenous peroxidase activity. Next, 50 μL aliquot of polymer enhancer was added to each section, which was followed by incubation of the sections at 37° C for 20 min. Secondary antibody (50 μL, Abcam, ab205718, goat anti-rabbit, 1 : 2000) was then added dropwise to each section, and incubated at 37° C for 30 min. The sections were exposed to diaminobenzidine reagent, and hematoxylin counterstaining. The brown color represented positive expression.

### ChIP assay

The ChIP assay was performed accordance with the manufacturer's instructions of the ChIP assay kit (Upstate Biotechnology, Lake Placid, NY, USA). Firstly, the cells were incubated with 50 μL Protein A/G PLUS-Agarose (sc-2003) for a duration of 30 min at the temperature 4° C, followed by a centrifugation for 5 min at 4° C. The supernatant was incubated with the primary antibodies to EED (1 : 100, PA5-34430, Invitrogen) and H3K27me3 (1 : 100, ab6002, Abcam) at 4° C overnight, followed by incubation with 50 μL protein A/GPLUS-Agarose (sc-2003). The beads were collected by centrifugation at 12,000 rpm for 2 h at 4° C. The beads were then washed twice with 1 mL of dissolution buffer high salt (sc-45001), followed by rinsing four times with wash buffer (sc-45002). The beads were further resuspended in 400 μL of elution buffer (sc-45003). After decross-linking, the beads were removed by centrifugation. The supernatant was further incubated overnight at 67° C, followed by centrifugation at 10,000 rpm for 3 min to with the purpose of removing any residual beads. The supernatant was harvested. To separate DNA, the supernatant was extracted once with 500 μL of phenol/chloroform/isoamyl alcohol (25 : 24 : 1), vortexed thoroughly and centrifuged at 14,000 rpm for 3 min. The aqueous phase from the previous step was preserved, and the organic phase was further extracted once with 100 μL of 10 mM Tris, 1 mm ethylenediaminetetraacetic acid (EDTA), pH 8.1 (TE) and pool water, and 600 μL of chloroform/isoamyl alcohol was employed to extract the aqueous phase.

### CCK-8 assay

Cells (2 × 10^3^) were seeded into 96-well plates. An enzyme-linked immunosorbent assay plate reader (Thermo Labsystems, Helsinki, Finland) and CCK-8 kit (Dojindo Laboratories, Kumamoto, Japan) were employed to measure absorbance of these cells at 450 nm at different time points according to the manufacturer's instructions. The results recorded were from three independent experiments, each with three replicates.

### Colony formation assay

The transfected cells of each group were seeded in 6-well plates at a cell density of 10,000 per well, and were cultured for 2 weeks, and after which the cells were stained with 0.5% crystal violet for 30 min. Excessive staining solution was washed twice with phosphate buffer saline (PBS), and the number of colonies was counted with a computer software.

### Transwell assay

Matrigel (YB356234, Shanghai Yubo Biotech, China) which was stored at -80° C was taken out and equilibrated at 4° C overnight to melt into liquid. Then, 200 μL Matrigel gel was diluted with 200 μL of serum-free medium. 50 μL of diluted Matrigel was added to upper chamber of each Transwell plate, which was followed by incubation for 2 - 3 h until the gel became solid. Cells were detached, and cell suspensions were prepared in serum-free medium. Next, 200 μL of cell suspension was added to the upper chamber of each well, and 800 μL of 20% FBS-conditioned medium was added to the lower chamber, followed by incubation at 37° C for 20 - 24 h. Next, the Transwell plate was rinsed twice with PBS, and fixed in formaldehyde for a duration of 10 min. The cells were then stained with 0.1% crystal violet and left to stand at room temperature for 30 min. The cells on the upper surface were wiped with a cotton ball, the remaining cells were observed, photographed and counted under an inverted microscope.

### Me-RIP assay

The total RNA was isolated from GC cells by employing the TRIzol method, and the mRNA from the total RNA was isolated and purified using the PolyATtract® mRNA Isolation Systems (A-Z5300, A&D Technology Corporation, Beijing, China). The anti-m6A antibody (1 : 500, ab151230, Abcam) or anti-IgG antibody (ab109489, 1 : 100, Abcam) was added to the IP buffer (20 mM Tris pH 7.5, 140 mM NaCl, 1% Nonidet P-40, 2 mM EDTA), which was then incubated with protein A/G magnetic beads for 1 h for binding. The IP buffer containing the ribonuclease inhibitor and protease inhibitor was incubated with the purified mRNA and magnetic bead-antibody complex overnight at 4° C. The RNA was then eluted with an elution buffer and purified by a phenol-chloroform extraction, and analyzed by RT-qPCR [15].

### *In vivo* tumor xenograft model

The lentiviral interference vector pSIH1-H1-copGFP (sh-, Cat. No. 8619936, BioVector plasmid vector strain cell gene collection center, Beijing, China) and the lentiviral overexpression vector pLV-EGFP-N (oe-, Cat. No. VL3211, Beijing Yingmao Shengye Biotechnology Co., Ltd., Beijing, China) were employed to construct a lentivirus-based EED gene interference vector and a CDCP1 gene overexpression vector. A stably infected human GC MGC-803 cell line was obtained.

Eighteen BALB/c male nude mice (Shanghai Slac Laboratory Animal Co., Ltd., Shanghai, China, aged 4 - 18 weeks, weighing 18 - 22 g) were selected for this study. A subcutaneous GC xenograft model of MGC-803 cells was established in nude mice. The mice were then randomly divided into 3 equal groups of 6: sh-NC + oe-NC, sh-EED + oe-NC, and sh-EED + oe-CDCP1. Tumor growth was observed and recorded by measuring tumor long diameter (A) and short diameter (B) using a vernier caliper after inoculation. Tumor volume was calculated using the formula tumor volume = (A × B^2^)/2. Nude mice in each group were euthanized by cervical dislocation on day 30. Tumor tissues were removed, photographed and weighed.

### Statistical analysis

SPSS version 19.0 (IBM SPSS Statistics, Chicago, IL, USA) was employed for the statistical analysis of the study. The measurement data with normal distribution and homogeneous variance were expressed by mean ± standard deviation. The paired data with normal distribution and homogeneous variance between two groups were compared using the paired *t* test, and the unpaired data using the unpaired *t* test. Data comparison among multiple groups was performed using one-way analysis of variance (ANOVA) with Tukey's post hoc test. Furthermore, data analysis between groups at different time points was performed using two-way ANOVA or repeated-measures ANOVA with Bonferroni post hoc test. Pearson’s correlation analysis was employed to analyze the correlation between miR-338-5p and EED expression in GC tissues. In addition, the Kaplan-Meier method was used to investigate the relationship between high and low expressions of EDD in GC tissues and the overall survival and disease-free survival (log-rank test). The difference was statistically significant at *p* < 0.05.

### Availability of data

The datasets generated/analyzed during the current study are available.

### Consent for publication

Consent for publication was obtained from the participants.
